# Combination of Quercetin or/and siRNA-loaded DDAB-mPEG-PCL hybrid nanoparticles reverse resistance to Regorafenib in colon cancer cells

**DOI:** 10.1186/s12906-022-03787-8

**Published:** 2022-12-27

**Authors:** Shabnam Shahidi, Kobra Rostamizadeh, Mojtaba Fathi, Keivan Nedaei, Ali Ramazani

**Affiliations:** 1 Department of Clinical Biochemistry, School of Medicine, Zanjan University of Medical Sciences, Zanjan, Iran; 2 Department of Pharmaceutical Biomaterial, School of Pharmacy, Zanjan University of Medical Sciences, Zanjan, Iran; 3 Zanjan Pharmaceutical Nanotechnology Research Center, School of Pharmacy, Zanjan University of Medical Sciences, Zanjan, Iran; 4 Cancer Gene Therapy Research Center, Zanjan University of Medical Sciences, Zanjan, Iran; 5 Department of Biochemistry and Genetics, Qazvin University of Medical Sciences, Qazvin, Iran; 6 Department of Medical Biotechnology, Faculty of Medicine, Zanjan University of Medical Sciences, Zanjan, Iran; 7 Department of Pharmaceutical Biotechnology, Zanjan University of Medical Sciences, Zanjan, Iran

**Keywords:** Colorectal cancer, Regorafenib, α5β1integrin, Quercetin, DDAB mPEG-PCL

## Abstract

**Background:**

Colorectal cancer (CRC) is the second leading cause of cancer death. Although Regorafenib showed survival benefits in patients with CRC, reports imply the recurrence of malignant phenotype resulting from chemotherapy. Evidence demonstrated that a5β1 integrin plays an important role in the Regorafenib treatment, which, may be led to resistance. In this study, the effects of /siRNA or/ and Quercetin loaded DDAB-mPEG-PCLnanoparticles could reverse this resistance phenotype in colon cancer cells in vitro.

**Methods:**

Regorafenib-resistant Ls-180 colon cancer cell line was developed by long-term exposure to Regorafenib. Quercetin and Regorafenib were separately encapsulated into mPEG-PCL micelles through the nano-precipitation method and characterized by DLS. Optimized doses of Quercetin and Regorafenib were used for combination therapy of resistant cells followed cytotoxicity study using MTT. Gene expression levels of the β1 subunit of integrin were determined by the real-time method of RT-PCR.

**Results:**

Developed Regorafenib resistant LS-180 showed to have Regorafenib IC50 of 38.96 ± 1.72 µM whereas IC50 in non-resistant cells were 8.51 ± 0.29 µM, which meaningful was lower statistically compared to that of a resistant one. The β1 mRNA level of whole α5β1 integrin was significantly higher in the resistant cells compared to those of non-resistant ones. Gene expression levels in each siRNA-loaded nanoparticle and Quercetin-loaded one were lower than that in mock experiments. Finally, when these two types of nanoparticles were used to treat resistant cells, gene expression decrease of integrin indicated a greater effect that could be capable of reverse resistancy.

**Conclusion:**

Results of this study demonstrated another confirmation of involving integrins in cancer resistance following chemotherapy using Regorafenib. Also, it indicated how using siRNA targeting integrin could enhance the plant derivatives like Quercetin effects to reverse resistance in vitro.

## Background

Despite the continuous achievements in cancer therapy, colorectal cancer (CRC) has become one of the top three leading causes of cancer-related deaths worldwide [1]. Currently, primary colorectal lesions are surgically removed. For more advanced lesions, systemic treatments, including chemotherapy, radiation therapy, and immunotherapy are needed [2]. Regorafenib (Stivarga) is an oral last-line chemotherapeutic agent in metastatic patients (mCRC), which could suppress tumor invasion, angiogenesis, and proliferation by alteration in oncogenes [3]. One of the oncogenic proteins is vascular endothelial growth factor (VEGF), which mainly regulates tumor angiogenesis. Regorafenib as a “VEGFR trap” inhibits multiple receptor tyrosine kinase (RTK) involved in tumor angiogenesis [4]. Also, Regorafenib could trigger both extrinsic and intrinsic apoptosis pathways [3]. Regorafenib has a demonstrated effect on CRC patients in comparison to similar drugs. However, resistance to treatment remains the major challenge in successful CRC treatment. Besides, the 5-year survival rate is less than 15% in metastatic patients (mCRC) [1]. It seems that the failure of effective anticancer drugs in most metastatic cancer patients depends on the cancer stem cells [5]. There is significant heterogeneity in the tumor microenvironment (niches) due to different subsets of stromal cells. CSCs are the small subpopulation of the tumor niches [6]. Relapse-initiating capabilities of these cells, along with invasive and metastatic proficiency, make them responsible for resistancy [7].

Another key component of the tumor niche is the Integrin, which is a bridge between cell and their microenvironment [8]. Integrin expression maintains the stemness of CSCs and resistance to therapy by directing fate decisions of multiple stem cell functions [9]. In addition, integrin contributes to the evolution of the malignant phenotype [10, 11]. Among them, alpha-5-beta-1 (α5β1) receptor is mostly up regulated in several cancers, including colorectal cancer [12]. Following the inactivation of β1 integrin, the self-renewal ability of cancer stem cells was significantly reduced [13], resulting in disruption of the polymerization and assembly of actin proteins [14]. In one study, β1 antagonists could indicate high potency against drug resistance [15]. Since a plethora of mechanisms has been driving by integrin, targeting one or even more integrin alone will not have curative effects [9]. Instead, combining anti-integrin drugs with phytochemical drugs seems to be a better treatment option. The biological impact of dietary phytochemicals such as flavonoids has been studied lots of time in cancer therapy [16]. Quercetin is the most familiar flavonoid in daily diet, which is enriched within Quercetin-rich plant or food supplements like grapes, berries [17]. It has been indicated that Quercetin significantly prevents the cell cycle and promotes apoptosis along with having a down-regulation effect on the VEGF signaling pathway [18, 19].

On the other hand, the ineffectiveness of existing treatments depends on the limitations of traditional drug delivery systems. High dose, instability, low bioavailability, poor solubility, insufficient time for absorption are some of them [20]. Nowadays, several studies are ongoing to promote the delivery and selective distribution of drugs to the desired location to achieve maximum effectiveness [21]. Indeed, hybrid nanoparticles (HNP) is the latest generation of nanocarriers carrying both core and shell structures with capability of loading hydrophilic and hydrophobic agents. Combinatorial drug deliveries without affecting the drug release profile is another capacity of these carriers [22]. The amine groups in the structure of Didodecyldimethylammonium bromide (DDAB) could adsorb any negative charge compounds like nucleic acids by having positive charges [23]. Small interfering RNA (siRNA) is one of nucleic acid therapeutics, which has shown successful applications due to affecting the post-translational modification and reducing the risk of teratogenicity [24]. As a matter of fact, despite the therapeutic potential of siRNAs, their renal filtration and reticuloendothelial (RES) up-taking do not allow uncoated administration for down-regulation of a targeted gene [25].

In this study, HNPs consist of a biodegradable polymer (mPEG-PCL and DDAB cationic lipid were constructed followed by encapsulation of quercetin in the core of the HNPs. Then, a5B1 siRNA was coated on the shell of the nanoparticle. HNPs carrying both quercetin and siRNA targeting a5B1 led to decrease of integrin beta-1 subunit and subsequent diminish of cell viability in colon cancer cells.

## Materials and methods

### Materials

The human colon cancer LS-180 cell line (ATCC® CL-187™) was purchased from Pasteur Institute (Tehran, Iran). They were cultured in RPMI-1640 supplemented with 10% fetal bovine serum, 1% penicillin (100U/mL), and streptomycin (100 µg/ml) (Gibco, NY, USA) at 37 °C in a 5% CO2 humidified incubator. To establish drug-resistant cells, LS180-colon cells were treated with constant doses of 40µM Regorafenib for three days, followed by recovery for five days. This treatment/recovery cycle repeated a total of 4 times [26]. In addition, integrin-β1 siRNA was synthesized by Microsynth AG (Switzerland) while Eurofins Genomics (Germany) synthesized the control siRNA. The sequence of integrin-β1 siRNA and control siRNA are shown in Table 1. Moreover, mPEG-PCL di-block copolymer was synthesized using the ring-opening polymerization of caprolactone in the presence of mPEG according to our previously published reports [27]. Regorafenib (Santa Cruz, CAS: 755037-03-7) and Quercetin (Sigma Aldrich, St. Louis, USA, CAS, 117-39-5) were purchased locally. In addition, Vivaspin filter with an MWCO of 10 kDa was used. Other chemicals were of analytical grade.

**Table 1:** The sequence of Integrin-β1 siRNA and control siRNA

**β1-integrin siRNA**	**sense**	**5’-UAGAUAUCUCGCGUCAUACdTdT-3’**
	anti-sense	5’-GUAUGACGCGAGAUAUCUAdTdT-3’
**control siRNA**	Sense	5’-UAGAUAUCUCGCGUCAUACdTdT-3
	anti-sense	5’-GUAUGACGCGAGAUAUCUAdTdT-3’

## Preparation of nanoparticles

### Preparation of Regorafenib-loaded nanoparticles

Reg/DDAB mPEG-PCL nanoparticles (RDMP) were prepared by a single-step nanoprecipitation method. Briefly, a stock solution of DDAB was prepared at a concentration of 1 mg/mL in distilled water. Regorafenib (0.05 mg) and the mPEG-PCL di-block copolymer (2.5 mg) were co-dissolved in acetonitrile. Then, after preparation a mixture of 400 µL R/mPEG-PCL and100 µL DDAB stocks 4000 µL deionized water was added. Volume ratio of aqueous to the organic solution was adjusted to be 10:1. Then, the mixture was sonicated in a capped glass vial for 6 min, using a bath sonicator at a frequency of 50 Hz and power of 350 W (Euronda, Eurosonic 4D, Italy). Using Vivaspin columns, centrifugation was performed at 3000 rpm for a minimum of 30 min to remove solvent [23].

### Preparation of Quercetin-loaded nanoparticles

Q/DDAB mPEG-PCL nanoparticles (QDMP) were prepared by a two-step nanoprecipitation method. Briefly, Quercetin (0.5 mg) and mPEG-PCL di‐block copolymer (4 mg) were co-dissolved in 2 ml of acetone and sonicated for 10 min using a bath sonicator at a frequency of 50 Hz and power of 350 W. This solution was then poured drop‐wise by a syringe (G = 22) into 18 ml of distilled water (carrying 2 ml DDAB stock solution) under vigorous stirring. Finally, decanting of solvent was done by centrifugation as described above [28].

## Characterization of HNPs

### Determination of size and zeta potential of HNPs

The particle size and zeta potential of both RDMP and QDMP micelles were determined by a Zeta sizer Nano ZS (Malvern Zeta sizer Nano ZS90). The polydispersity index of the nanoparticles was simultaneously measured for determination of the particle size. All measurements were carried out in triplicate.

### Determination of encapsulation efficiency and drug loading of HNPs

Encapsulation efficiency (EE) and Drug loading (DL) of the RDMP and QDMP were determined using calibration curves via spectrophotometric method. Briefly, both Regorafenib and Quercetin quantities in the supernatants of prepared RDMP and QDMP were measured with a UV-Vis spectrometer (Madison, GENESYS™ 10 S; Thermo Fisher Scientific, USA) at 261 and 384 nm, respectively and their concentrations obtained from corresponded calibration curves. For obtaining the total amount nanoparticles without the drug (NP), the nanoparticles were dried and weighed after synthesis.

The following formulas were applied to determine encapsulation efficiency (EE%) and drug loading (DL%)

$$\mathrm{EE}\%\;=\;(\mathrm{amount}\;\mathrm{of}\;\mathrm{encapsulated}\;\mathrm{drug}\;\mathrm{in}\;\mathrm{free}\;\mathrm{form})/(\mathrm{Total}\;\mathrm{amount}\;\mathrm{of}\;\mathrm{free}\;\mathrm{drug})\;\;\times\;100$$

$$\mathrm{DL}\%\;=\;(\mathrm{amount}\;\mathrm{of}\;\mathrm{encapsulated}\;\mathrm{drug}\;\mathrm{in}\;\mathrm{free}\;\mathrm{form})/(\mathrm{Total}\;\mathrm{amount}\;\mathrm{of}\;\mathrm{free}\;\mathrm{drug}\:+\:\mathrm{amount}\;\mathrm{of}\;\mathrm{NP})\;\times\;100$$

Standard curves were plotted using concentrations (x-axis) against absorbances (y-axis). The LOQ, Limit of Quantification, and the LOD, Limit of detection, were also determined according to the following formula:

$$\mathrm{LOD}\;=\;(\mathrm{SD}/\mathrm{Slope})\;\times\;3.3$$

$$\mathrm{LOQ}\;=\;(\mathrm{SD}/\mathrm{Slope})\;\times\;10$$

### Gel retardation assay

To determine optimum concentration of siRNA load on the surface of the HNP, 52 ng (constant) of beta − 1 targeting siRNA was mixed with different ratios of HNPs to construct HNP/siRNA complexes with different polymer amine nitrogen/nucleic acid phosphate (N/P) ratios. Therefore, different N/P ratios of HNP/siRNA complexes were mixed with 2 µL loading buffer, loaded on 2% agarose gel, and separated by electrophoresis at 100 V for 20 min. The gel was stained with gel red, and the bands corresponding to HNP/siRNA complexes were visualized under UV light transilluminator (Uvitec 1QB-UK). N/P ratios of HNP concentrations were calculated according to the following formula:

$$\mathrm{The}\;\mathrm{volume}\;\mathrm{of}\;\mathrm{NP}\;(\mathrm\mu{L})\;=\;((\mu{g}\;\mathrm{siRNA})\;\times\;(\mathrm{nmol}\;\mathrm{phosphate}\;\mathrm{per}\;\mu{g})\;\times\mathrm{desired}\;(\mathrm N/\mathrm P))/\;(\mathrm{nmol}\;\mathrm{cationic}\;\mathrm{lipid}\;\mathrm{per}\;\mu{L})$$

It should be explained that, one microgram of siRNA contains 3 nanomoles of phosphate and one microgram of DDAB contains 0.634 nanomoles of nitrogen as cationic lipids. Therefore, different N/P ratios require different HNP volumes.

### Characterization of HNP/siRNA complexes

The particle size and zeta potential of both RDMP/siRNA and QDMP/siRNA complexes were also characterized by a Zeta sizer Nano ZS (Malvern Zeta sizer Nano ZS90). To determine siRNA encapsulation efficiency, RDMP/siRNA and QDMP/siRNA complexes were prepared at optimum N/P ratios. After one hour of incubation at RT, samples were centrifuged at 3000 rpm for 30 min. Then, the amount of free siRNA of the precipitant was analyzed by Nanodrop at a wavelength of 260 nm. SiRNA Encapsulation efficiency (EE) were calculated according to the following formula:

$$\%\mathrm{EE}=\;((\mathrm{total}\;\mathrm{amount}\;\mathrm{siRNA}-\;\mathrm{free}\;\mathrm{amount}\;\mathrm{siRNA})/\mathrm{total}\;\mathrm{amount}\;\mathrm{siRNA})\;\times\;100$$

### Determination of cell viability

The growth-inhibitory activity of the free drugs (Regorafenib and Quercetin), RDMP and QDMP nanoparticles, and also HNP/siRNA complexes (RDMP/siRNA and QDMP/siRNA) on colon cancer cells were assessed by using MTT. Both resistance and non-resistance LS-180 cells seeded in 96 wells plates at a seeding density of 15 × 10 ^3^ cells/well. After pre-incubation of 48 h, old media was replaced with fresh serum-free media containing various concentrations of free drugs, RDMP, QDMP, and their complexes with siRNA. After 24 h post-transfection, 40 µL of MTT (5 mg/mL in PBS) was added to each well. Then, the optical density (OD) values were measured at 570 nm using a Stat Fax- 2100 micro plate reader (Awareness Technology, USA) after 3 h later. Results tested in triplicate. Cell viability was calculated by: (mean OD treated well [− blank])/ (mean OD control well [− blank]) × 100. Finally, three lower doses than IC_50_ including IC_10_, IC_20_, and IC_30_ used for the following Quercetin treatment.

The IC_50_ (half maximal inhibitory concentration) values are also obtained from the cell- viability graph.

### In vitro transfection

Regorafenib-resistant Ls-180 colon cancer cell lines were treated with different doses of the free drugs (Regorafenib and Quercetin), and their partners as a nanoparticles or HNP/siRNA complexes.

### In vitro gene silencing

After being cultured and treated, total RNA from the cells in the groups and subgroups (Table 2) were extracted using the TrizolEX Reagent (DNAbiotech Co. I.R. Iran), according to the manufacturer’s protocol, followed by DNase I treatment. The quantification of extracted total RNA was measured using a NanoDrop ND-1000 spectrophotometer (Wilmington, DE, USA). The RNA purity estimated from the OD 260/280 and the OD 260/230 ratios. Then, cDNA synthesis was performed by reverse transcriptase with M-MLV First Strand kit (Favorgen Biotech Corp, Taiwan), according to the manufacturer’s instructions. Reactions incubated in a FlexCycler2 system (Biometra GmbH, Germany). Finally, quantitative real-time PCR (qRT-PCR) was done using BioFact™ 2X Real-Time PCR Master Mix (for SYBR Green I; BIO FACT, South Korea). Beta-actin (βact) was utilized as the internal control to quantitatively determine the messenger RNA (mRNA) expression levels of β1-integrin. Moreover, no template controls (NTC) were included for the target gene in each run.

**Table 2:** Treatment groups and doses

**Groups**	**Subgroups and definition**
**A) Regorafenib**	A_1_:40µM regorafenib A_2_: Nano-particles containing 40 µM regorafenib (RDMP) A_3_: Nano-particles containing 40 µM regorafenib (RDMP) and 156nM siRNA
**B) Quercetin**	C_1_: 8.2 µM quercetin = IC_10_ C_2_: 28.7 µM quercetin = IC_20_ C_3_: 65.7 µM quercetin = IC_30_
**C) Regorafenib/ Quercetin**	B_1_: 40µM regorafenib and 8.2 µM quercetin B_2_: 40µM regorafenib and 28.7 µM quercetin B_3_: 40µM regorafenib and 65.7 µM quercetin
**D) QDMP /siRNA**	D_1_: Nano-particles containing 8.2 µM quercetin (QDMP) and 11 nM siRNA D_2_: Nano-particles containing 28.7 µM quercetin (QDMP) and 35.8 nM siRNA D_3_: Nano-particles containing 65.7 µM quercetin (QDMP) and 88 nM siRNA
**E) NP control**	E_1_: Nano-particles containing 156nM siRNA E_2_: 156 nM non-targeted-siRNA
**F) Control**	F_1_; cancerous cells without treatment F_2_; resistant cancerous cells without treatment

## Statistical analyses

Kolmogorov-Smirnov test was applied to assess the normality of the distribution of the variables. For assessing the differences among the subgroups, one-way ANOVA was used followed by a posthoc test. The differences between the two variables were measured by an independent sample t-test. For statistical analysis of data, SPSS Version 20 (IBM Corporation, Armonk, NY, USA) was applied, and graphs were constructed by Graph Pad Prism® 8 software (Graph Pad Software Inc., San Diego, CA, USA). All data were expressed as mean ± SD. A value of *p* < 0.05 was considered statistically significant. Every experiment was repeated at least three times independently.

## Results

### Establishment of the resistant cell line

**Fig. 1:**
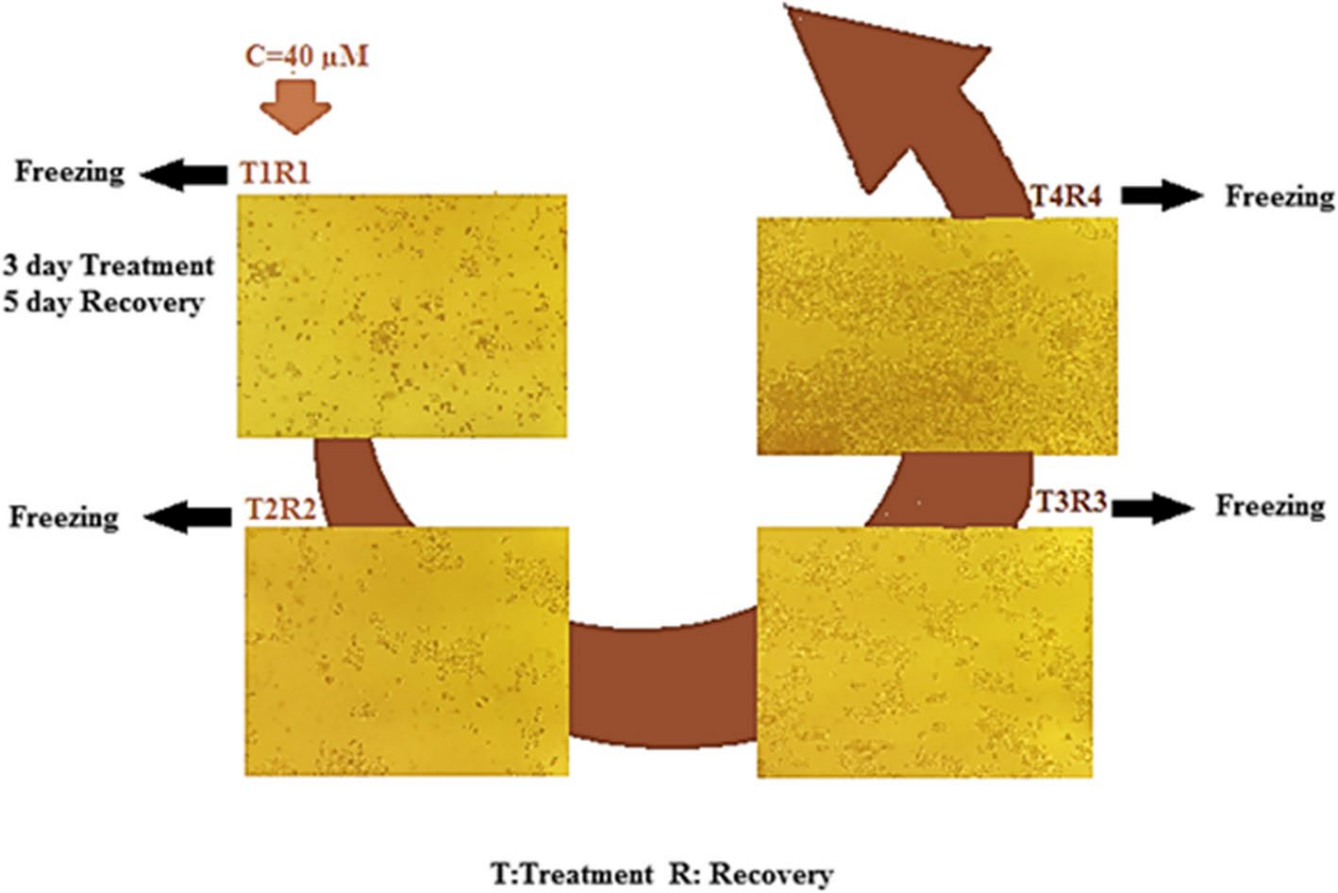
Cell morphology of Regorafenib-resistant LS-180 cell line at the end of each cyclic is shown. The cells were resistant during four cycles using a fixed dose of 40 µM Regorafenib. As we approached the last cycles, the cells became less sensitive to the drug. To the extent that the behavior of cells over the fourth cycle was no different from that of cells that grew in drug-free media. T: treatment; R: recovery

**Fig. 2:**
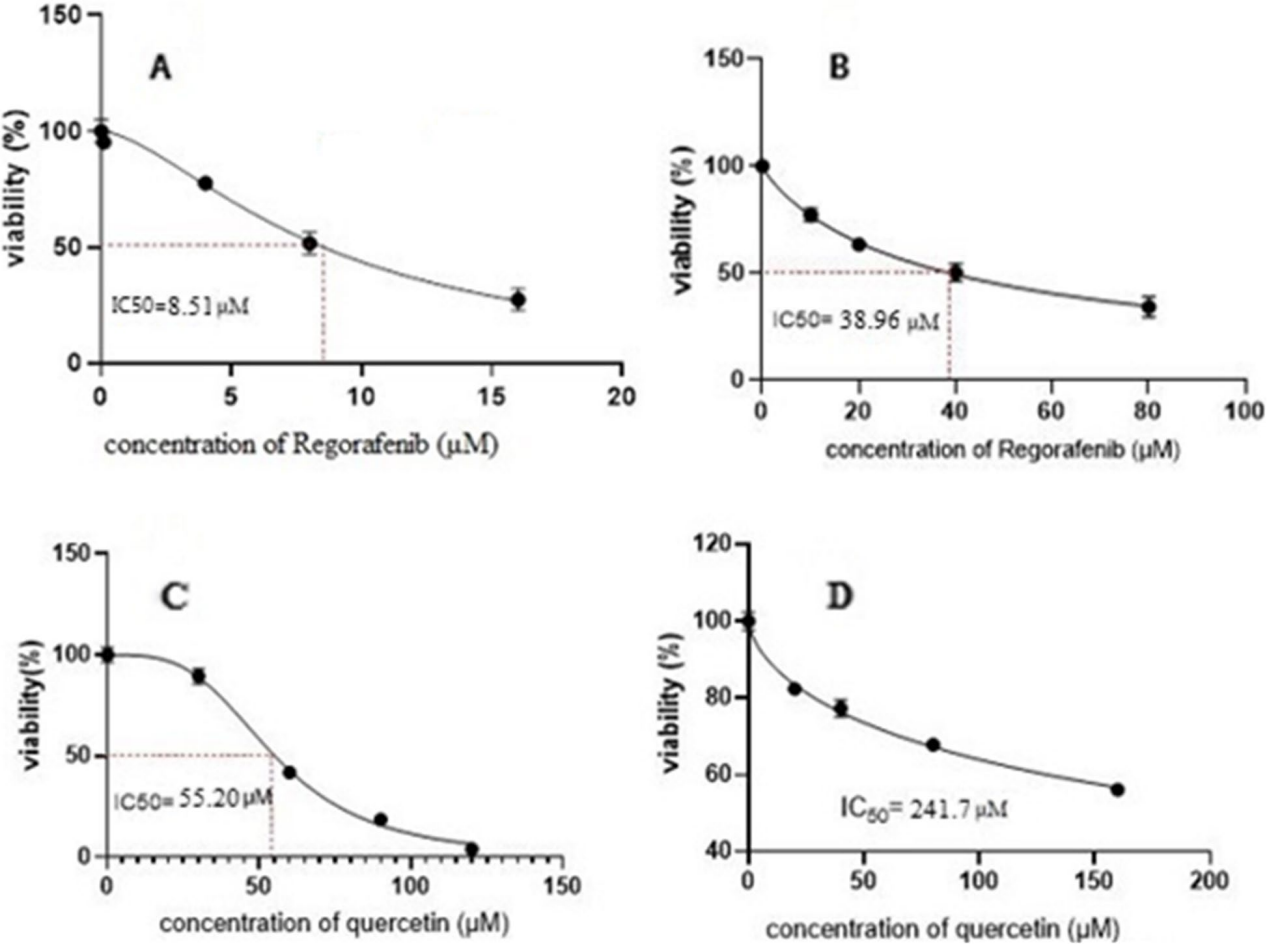
** A** Dose- response survival of LS-180 cells after exposure to increasing concentrations of Regorafenib for 24 h; **B** Dose- response survival of resistance LS-180 cells after exposure to increasing concentrations of Regorafenib for 24 h; **C** Dose- response survival of LS-180 cells after exposure to increasing concentrations of Quercetin for 24 h; **D** Dose- response survival of resistance LS-180 cells after exposure to increasing concentrations of Regorafenib for 24 h

Parental cell lines were growing in the presence of a constant higher dose (40 µM) of Regorafenib. The cellular morphological structures of LS-180 cells were observed at the end of the third day after transfecting drug-enriched medium and also at the end of the recovery period. The difference in cell confidence could also be due to the stressful conditions in which cells thrive (Fig. 1 ). Evaluation of the half-maximal inhibitory concentration (IC_50_) as the most widely used and informative measure of a Regorafenib’s efficacy in both cell types. As shown in Fig. 2, IC_50_ in non-resistant cells for Regorafenib was 8.51 ± 0.29 µM (Fig. 2 A), but it increased 38.96 ± 1.72 µM in the resistant cells (Fig. 2B).

#### Effects of Quercetin on the viability of resistant LS-180 cell line

The inhibition of cell proliferation effect of Quercetin on LS-180 cells was examined by MTT assay. When the cells were treated by 0-120 µM Quercetin, IC_50_ was achieved to be 241.7 µM for resistant cells, while it tended to be significantly low up to 55.2 µM in non-resistance (*P* < 0.01) **(**Fig. 2**)**.

### In vitro cytotoxicity assay (MTT assay)

**Fig. 3:**
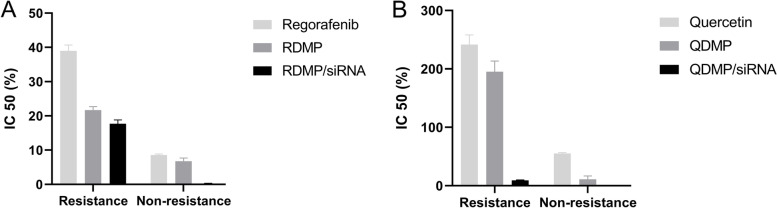
** A** The IC_50_ values of the Regorafenib group, including free drug (Regorafenib), RDMP, and RDMP/siRNA complex in both resistance and non-resistance cell lines. **B** The IC_50_ values of the Quercetin group, including free drug (Quercetin), QDMP, and QDMP/siRNA complex in both resistance and non-resistance cell lines. RDMP: Reg/DDAB mPEG-PCL nanoparticles; QDMP: Q/DDAB mPEG-PCL nanoparticles; siRNA; small interfering RNA

The cytotoxicity of drug-loaded nanoparticles (RDMP and QDMP) and HNP/siRNA complexes (RDMP/siRNA and QDMP/siRNA) on both resistance and non-resistance cell lines were also detected. According to the data, the IC_50_ values of the Regorafenib group decreased in the nano-drug delivery system compared to the free drug modes. Although; this reduction was not significantly different in the Regorafenib group between drug-loaded nanoparticle (RDMP) and free-drug (*P* = 0.343), but it was significantly different between HNP/siRNA complexes (RDMP/siRNA) and free-drug on both resistance and non-resistance cell lines (*P *< 0.05). The IC_50_ values of the Quercetin group significantly decreased compared to the free drug, either QDMP or QDMO/siRNA in both resistance and non-resistance cell lines (*P* < 0.01) (Fig. 3 ).

### Preparation and characterization of nanoparticles

**Fig. 4:**
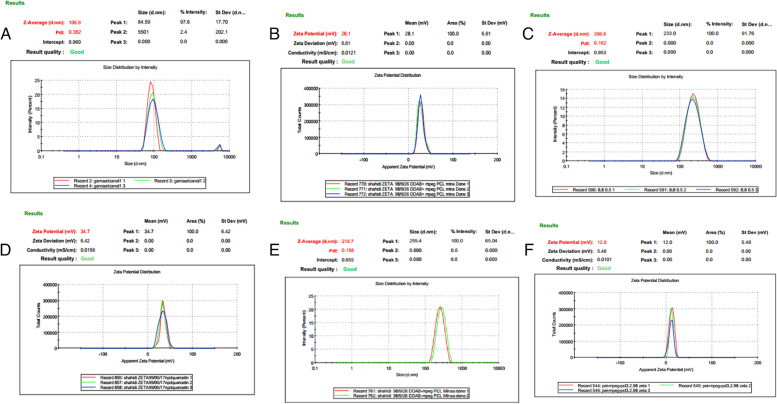
** A** Particle Size Distribution Measurements of RDMP by Laser Dynamic Light Scattering (DLS); **B** The Z-potential Measurements of RDMP by Laser Dynamic Light Scattering (DLS): **C** Particle Size Distribution Measurements of QDMP by Laser Dynamic Light Scattering (DLS); **D** The Z-potential Measurements of QDMP by Laser Dynamic Light Scattering (DLS); **E** Particle Size Distribution Measurements of QDMP/siRNA by Laser Dynamic Light Scattering (DLS); **F** The Z-potential Measurements of QDMP/siRNA by Laser Dynamic Light Scattering (DLS). RDMP: Reg/DDAB mPEG-PCL nanoparticles; QDMP: Q/DDAB mPEG-PCL nanoparticles; siRNA; small interfering RNA

The size of nanoparticles was measured by the dynamic light scattering technique (Fig. 4 ). As shown in Fig. 4 A and B, the z-average and zeta potential of RDMP micelles were found to be about 108.46 ± 2.13 nm and + 27.96 ± 0.25 mv, with their corresponding PDI being 0.337 ± 0.057. As shown in Fig. 4 C and D, the z-average and zeta potential of QDMP were found to be about 204.96 ± 2.09 nm and + 33.53 ± 1.98 mv, with their corresponding PDI being 0.162 ± 0.003. The drug loading and encapsulation efficiencies of both RDMP micelles were determined to be 2.22 ± 0.032% and 98. 63 ± 1.33% and it was for QDMP 7.61 ± 0.035% and 98.96 ± 0.45%, respectively. However, the obtained QDMP/siRNA micelles had a mean size of 217.60 ± 1.55 nm, the zeta potential of 12.06 ± 0.62 mv, PDI of 0.238 ± 0.084% 4E and 4 F).

### Regorafenib and Quercetin calibration curves

**Fig. 5:**
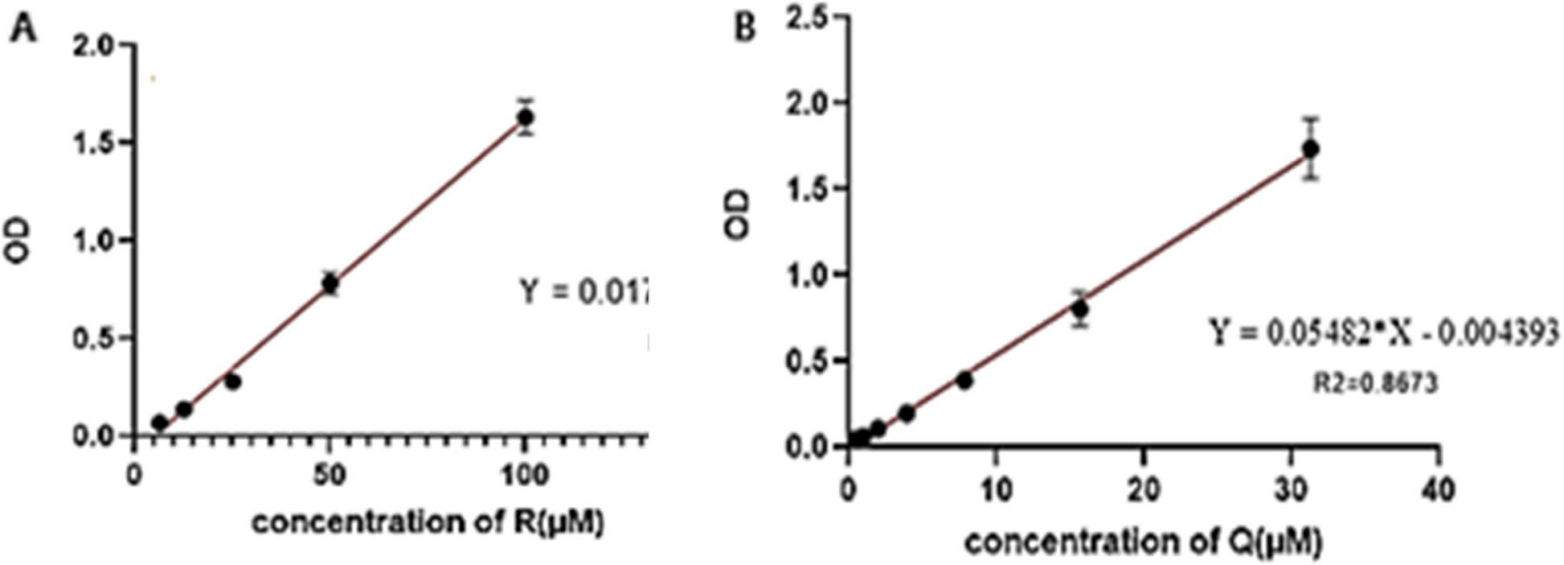
The X-axis shows the various concentrations of the diluted drugs (in mg/dL) and the Y-axis shows the degree of absorption reported as Optical density (OD) **A** Calibration curve in Quercetin (*n* = 3) with 95% CI. **B** Calibration curve Regorafenib (*n* = 3) with 95% CI

To investigate the performance of the drug loading method (DL) and to evaluate the encapsulation efficiency (EE) of RDMP and QDMP, specificity and selectivity were assessed by analyzing at least fifth independent samples. All samples were analyzed below the response of the LOQ. The LOQ of Regorafenib and Quercetin calibration curves were determined 85µM and 43µM, respectively. Also, the LOD of both Regorafenib and Quercetin calibration curves were 28µM and 14µM, respectively. Linearity was assessed using at least a five-point calibration curve (*n* = 3). With a weighting factor of 1 (*r*2 = 0.9446, *n* = 3), for Regorafenib and (*r*2 = 0.8673, *n* = 3), for Quercetin. The results are shown in Fig. 5.

### Preparation and characterization of HNPs/siRNA complexes

**Fig. 6:**
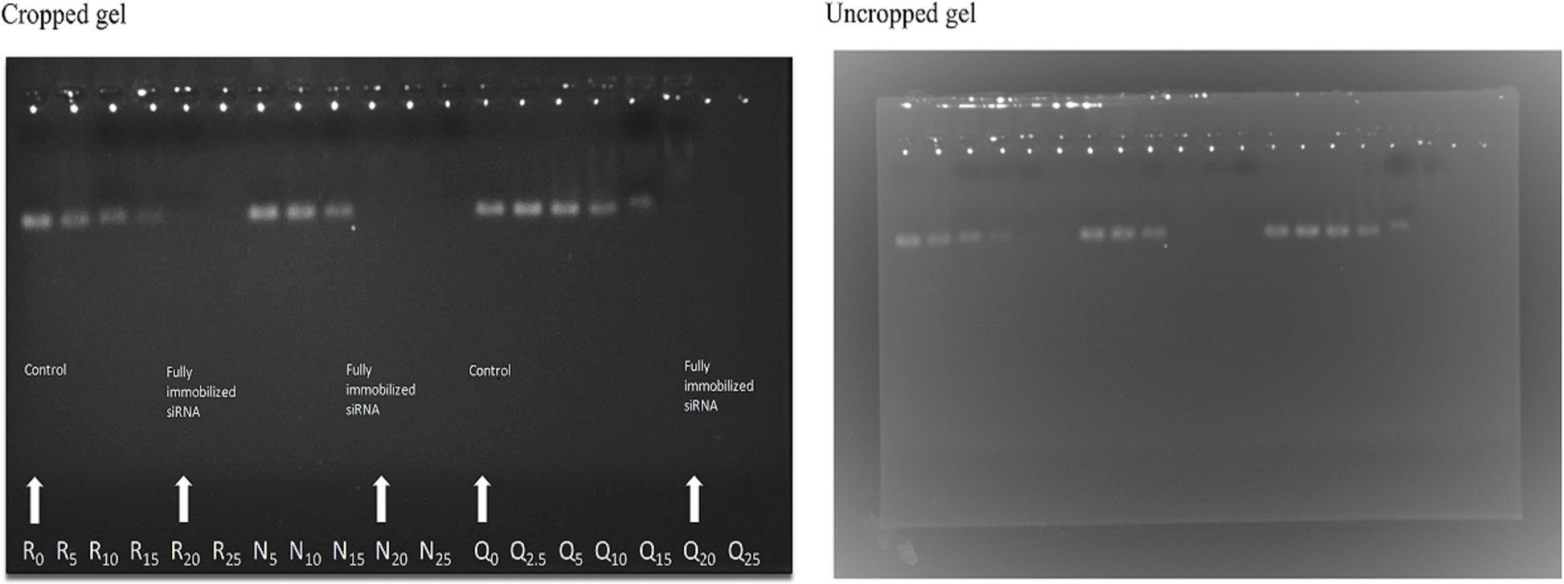
Agarose gel electrophoresis of free siRNA (1 µg) and QDMP/siRNA and RDMP/siRNA to determine the most effective ratio of N/P to fully immobilized siRNA. R: RDMP/siRNA, N: nanoparticle without drug Q: QDMP/siRNA

After centrifuging the prepared HNPs/siRNA complexes, the concentration of free siRNA in precipitant was measured, Using UV spectroscopy. The gel retardation assay was performed to study the binding capacity of HNP with siRNA. The results were shown in Fig. 6. According to Gel retarding analysis, at an N/P ratio of 20, both drug-loaded nanoparticles (RDMP and QDMP) and nanoparticles without the drug (NP) can fully immobilized siRNA. The further calculation, also indicated that the siRNA encapsulation efficiency was 97.65 ± 0.57%.

### β1 integrin expressions in the non-resistant cells compared with the resistant LS-180 cells

**Fig. 7:**
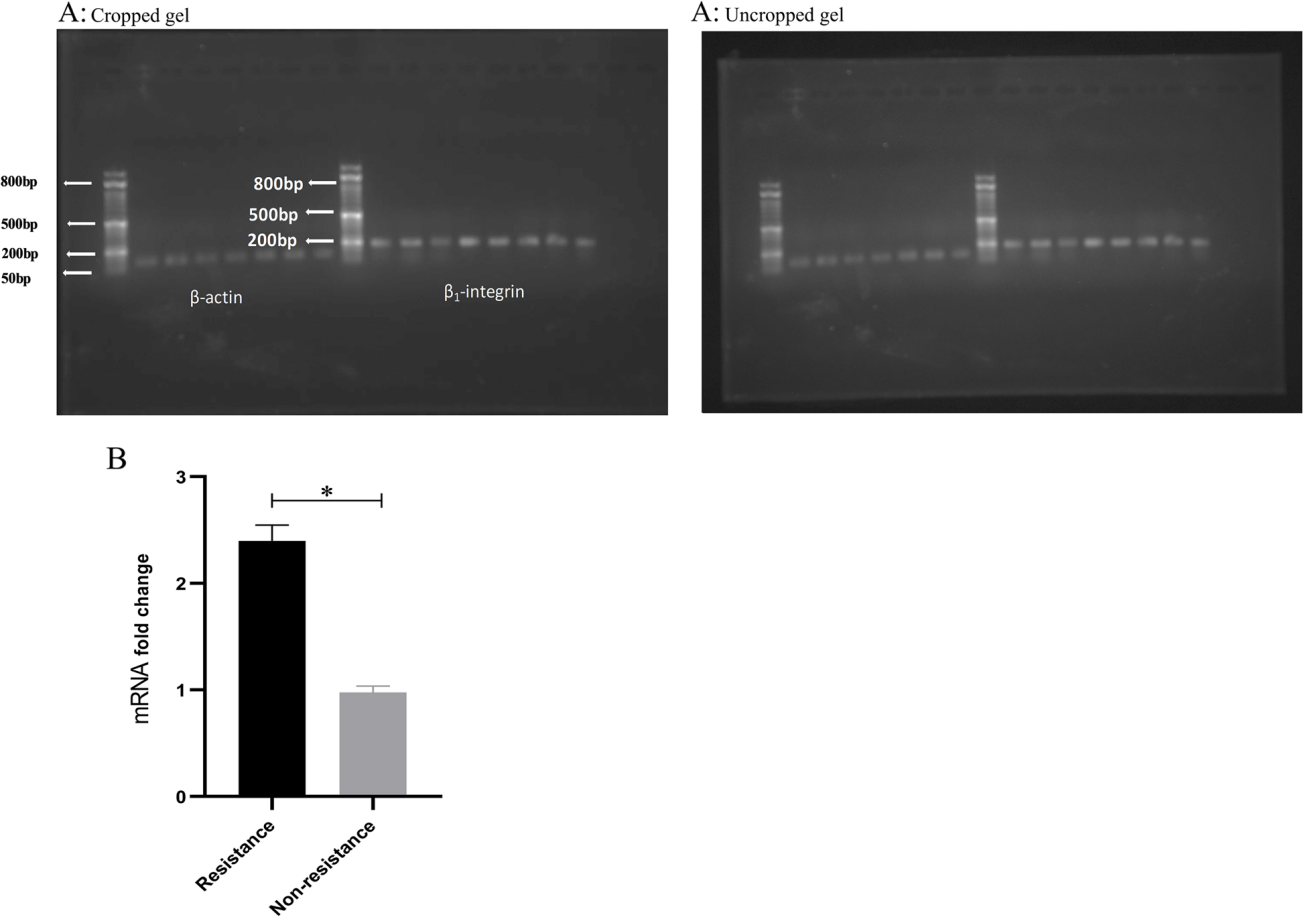
** A** Agarose gel electrophoresis of all real-time PCR products derived from β1-integrin & B-Actin. Left to right in the gel: 50 bp ladder, 28.7µM Quercetin, 65.7 µM Quercetin, 40µM Regorafenib, 40 µM RDMP, 40 µM RDMP and 156nM siRNA, and control cells; **B** β1 integrin expressions in the non-resistant cells compared with the resistant LS-180 cells

After RT-PCR performing, visualized gels indicated the expression of β1 normalized to the beta-actin as the internal control in different groups and subgroups **(**Fig. 7 A). According to the results, β1 mRNA expression showed 2.06 times higher value in the resistance compared with the nonresistant cells (*P* < 0.001) **(**Fig. 7B).

### β1 integrin expressions decreased in resistant LS-180 colon cells after combination therapy using Regorafenib and Quercetin

**Fig. 8:**
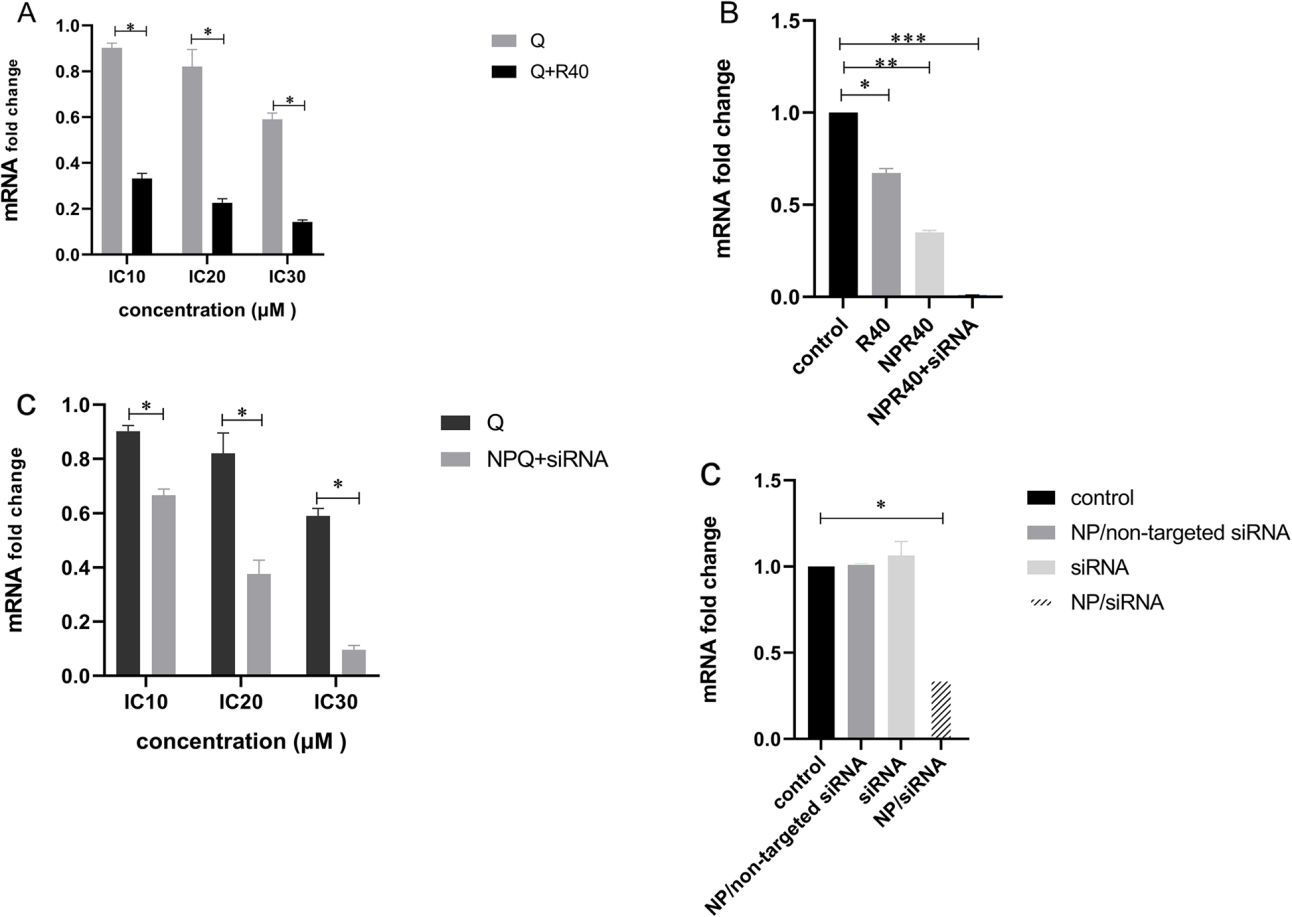
** A** Combination therapy using Regorafenib 40µM and increasing level of Quercetin could significantly down-regulate β1 integrin expression in resistant colon cancer cells (*p* < 0.01); **B** There was a significant difference not only between the Regorafenib subgroups but also between the Regorafenib group and untreated state (*p* < 0.05); **C** There was a significant difference between the subgroups of Quercetin and the QDMP/siRNA (*P* < 0.01). In the Quercetin group, a decrease in β1 integrin expression in resistant cells was significantly higher in IC_30_ than IC_10,_ IC_30_ than IC_20_ (*P* < 0.01), however, a significant decrease was not observed between IC_20_ and IC_10_ (*P* = 0.09). While, in the QDMP/siRNA group, inter-group comparison of treatment at all doses (IC_30_, IC_20_, and IC_10_) represented a significant decrease in β1 integrin expression; **D** No significant relationship was observed in siRNA subgroups. Except, NP/siRNA with untreated state (*p* = 0.02).NP: nanoparticle without drug. R: Regorafenib; Q: Quercetin; RDMP: Reg/DDAB mPEG-PCL nanoparticles; QDMP: Q/DDAB mPEG-PCL nanoparticles; siRNA; small interfering RNA

To show the combination therapy effect using Regorafenib and Quercetin on the α5β1 gene expression, β1 mRNA expression was measured using the RT-PCR method. The results have shown in **(**Fig. 8a). Taken together, the combination of 40µM Regorafenib with an increasing level of Quercetin up to 87 µM (IC_30_) could down-regulated synergistically β1 gene expression by at least 16.4%, in resistant cells.

### Regorafenib mediated down-regulation of β1 integrin expression in resistant LS-180 cells

The complex of RDMP/siRNA significantly reduced β1 integrin gene expression in comparison with NPR40 (RDMP) as well as with R40 at the same concentration of 40 µM (*P* < 0.05). Moreover, the results of analyses showed a significant inter-group relationship between R40, RDMP, and RDMP/siRNA, with the control group (untreated) (*p* < 0.01) (Fig. 8b).

### Quercetin mediated down-regulation of β1 integrin expression in resistant LS-180 cells

The LS-180 resistance cells were incubated with both QDMP/siRNA and free drug to evaluate the activity of QDMP/siRNA in gene silencing of β1 expression. In addition, untreated cells were considered as the control. When data analyzed, Quercetin down-regulated β1 integrin expression in resistant LS-180 cells compared with the control (*P* < 0.05) (Fig. 8c). Besides, at all doses (IC_30_, IC_20_, IC_10_), the complex of QDMP/siRNA showed a higher ability to inhibit the β1 integrin gene than the free drugs (Q) (*P* < 0.01).

## Discussion

Despite advances in therapeutic agents, colorectal cancer (CRC) remains the third leading cause of cancer death globally [1]. Integrins as heterodimer proteins expressed on endothelial cells. Not only, they involve in both inside-out and outside-in signaling pathways, but also they play roles in cell growth, survival, and migration during angiogenesis and apoptosis [29]. Given the broad roles of integrins as transmembrane proteins, they appear to play a fundamental role in the resistancy [30]. Since the signal transduction pathway of these proteins has in common with tyrosine kinase (RTK) receptors, some receptor tyrosine kinase (Bruton’s tyrosine kinase) inhibitors have been employed as therapeutic targets for integrins [30]. In this regard, and due to the increasing potency of drug resistance, studies on the role of integrins and their increased expression in various cancers have been rose. Previously, Carbonell et al. have made mention of increasing β1 integrin as the main factor in resistance to anti-angiogenic therapy [31]. To the best of our knowledge, this is the first in which Regorafenib has developed resistancy by β1integrin in LS-180-resistant colon cell line. The resistancy confirmed by the increased IC_50_ in resistant cells compared to non-resistant one (*P* < 0.05). The efficiency of this method has been evaluated in the study of Tong, J. et al. [26]. In line with the present study (IC_50_ of 38.96 µM in resistant cells and 8.51 µM in non-resistant ones), higher IC_50_ in resistant cells was found compared to the parental cells in Tong’s study [26]. On the other hand, in a study by Mirone et al. SW48 resistance cell lines have been generated by exposing to stepwise increasing concentrations of Regorafenib over 12 months. According to the Mirone study, the Regorafenib IC_50_ was 0.7µM, which increased more than 200-fold at the end of the resistance. Prolonged exposure and accumulation of mutations during this period can consider as the most cause of severe resistance to Regorafenib, in comparison to the present study [32], in which the resistant LS-180 colon cell line has shown increased levels compared to parental (*P* < 0.05). Carbonell, et al. have also revealed an eight-fold risen in bevacizumab-resistant glioblastoma in patients using flow cytometry. Given the differences in techniques, this value is acceptable. In addition, the IC_50_ amounts in the vast majority of nano-drug delivery systems, including drug-loaded nanoparticles (RDMP and QDMP) and HNP/siRNA complexes (RDMP/siRNA and QDMP/siRNA), reduced significantly relative to the free drug (*P* < 0.05). According to Javadi, et al. the decrease in IC_50_ concentration in synthetic nanoparticles compared to the free drugs indicated lower toxicity of synthetic nanoparticles as drug-delivery vehicles [33].

Although small interfering RNA (siRNA)-based gene therapy has provided a novel strategy for cancer therapy, high negative charge of siRNAs prevent them entry into cells [34]. Moreover, the siRNA sequence can activate the innate immune system, which boosts siRNA cleavage in the bloodstream [35]. Hence, the DDAB and MPEG-PCL hybrid micelles were prepared. Due to the presence of DDAB cationic liposomes in nanoparticles, siRNA could combine with it [36]. The efficiency of this siRNA delivery system has been proven in our previous report [23]. In the present study, the drug and siRNA were co-nano formulated this time, compared to the previous which drug-loaded nanoparticle and siRNA loaded nanoparticle were treated to the cells individually. It seems the amalgamation of anti-cancer drugs with nucleic base ones enhances efficacy compared to the mono-therapy approach. Overall, the purpose of loading anti-β1siRNA was to selectively distribute the drug at the target site [37]. DLS data show that the synthetic nanoparticles have the desired positive charge (+ 26.76). Lu et al. demonstrated that HNP delivered siRNAs could effectively inhibit the C26 colon cancer cell’s growth [38]. Also, they prepared their HNP by modifying the mPEG-PCL micelle with cationic DOTAP lipid. In contrast, we applied DDAB cationic lipid. Besides, the treatment of Regorafenib-resistant cell line LS-180 with either RDMP/siRNA or QDMP/siRNA, compared to NP/siRNA, significantly reduced the expression of the integrin β1gene (*P* < 0.05), while treating cells with non-targeted siRNA did not show such a result (*p* = 0.988). In Yongping et al. study, a significant decrease in target gene expression (Mcl 1 and Bcl-XL) was also seen [38]. To further characterize the utilized carrier, it is necessary to achieve the optimal mass ratio of HNP/siRNA (N/ P). Due to erythrocyte hemolysis after exposure to the positive charge, we minimized the N/P ratio. In a way, this ratio was 20, while Lu et al. has reached the mass ratio of ≥ 30 [38]. The difference may be attributed to the lower lipid content used in the synthesis of HNP or the higher siRNA content in complex formation. Overall, we have synthesized and characterized dual-Quercetin and siRNA nanoparticles (siRNA/QDMP). The size of dual nanoparticles is not significantly increased compared to nanoparticles encapsulated with Quercetin (QDMP) (217.60 ± 1.55 versus 204.96 ± 2.09 nm.). However, the zeta potential of siRNA/QDMP was 13.06 ± 0.92 mV in comparison to + 33.53 ± 1.98 mV for QDMP. In line with Hemati et al. the reduction in the zeta potential confirmed that the siRNA successfully incubated with the nanoparticle [39].

Quercetin is a natural flavonoid that plays various roles in cancer hallmarks. Meanwhile, its effect on angiogenesis, especially through molecules such as VEGF, links it to Regorafenib [40]. Over the last few decades, some studies have widely used Quercetin nanoparticles to ameliorate this flavonoid therapeutic application [39, 41] As far as we know, there is little information about Quercetin interaction with α5β1integrin, especially in colon cancer. In this study, we have investigated the co-delivery of Quercetin and siRNA nanoparticles in Regorafenib resistance colon cell line. According to data, Quercetin could down-regulate β1 integrin expression in resistant LS-180 cells. Moreover, in all treated doses, a comparison between gene silencing of Quercetin alone, and co-delivery of siRNA, QDMP for Regorafenib resistance cells demonstrated a significant reduction (*P* < 0.01).

Following the discovery of Regorafenib, some studies have performed on the combined effects of Regorafenib with other chemotherapeutic agents [42, 43]. On the other hand, the overuse and the combination of several chemotherapeutic drugs is a potential factor in drug resistance development [42]. Hence, in the last decade, the use of phytochemicals along with chemotherapy drugs has been developed to counteract drug resistance mechanisms [44]. We attempted to reduce drug resistance by the combination of Quercetin phytochemical with Regorafenib. For this purpose, constant concentrations of Regorafenib (40 µM) and increased doses of Quercetin were treated both individually and in combination. The results of combination therapy with Quercetin and Regorafenib showed a significant decrease in β1 expression compared to the free drug (*P* < 0.01). Given the steady concentration of Regorafenib in all treatments, the significant difference depends on the increasing concentration of Quercetin (*r*=-0.9, *P* = 0.002).

It should be noted that many studies have examined the synergistic effects of Quercetin in combination with a different chemotherapeutic agent [45]. According to these studies, Quercetin can boost the effectiveness and therapeutic index of chemotherapy drugs in-vivo and in-vitro [46]. In contrast, some revealed to Quercetin counteract other chemotherapeutic drugs. According to most published studies, these interactions often manifest themselves in high concentrations [47]. Due to differences in cell type and the potential of combined chemotherapeutic drugs, each study has achieved different concentrations. According to the present study, the 40 µM of Regorafenib combined with an increasing level of the Quercetin up to 87 µM has a greater effect on down-regulation of β1 integrin expression in resistance colon cancer cells. Similarly, the results obtained by Chuang-Xin demonstrated that the combination of 50 µM Quercetin with 200 µM of 5-fluorouracil (5-FU) synergistically induces apoptosis in esophageal cancer cells [48].

## Conclusion

The findings of present study demonstrated that integrins have an important role in cancer resistance following chemotherapy using Regorafenib. Moreover, it indicated siRNA by targeting integrin could enhance the efficacy of Quercetin in reverse cancer resistance in vitro.

## Data Availability

The data used to support the findings of this study are included within the article. Additional data or information can be requested by contacting the corresponding author.

## Abbreviations

CRC: Colorectal cancer
VEGF: Vascular endothelial growth factor
RTK: Receptor tyrosine kinase
HNP: Hybrid nanoparticle
DDAB: Didodecyldimethylammonium bromide
siRNA: Small interfering RNA
RES: Reticuloendothelial
RDMP: Regorafenib/ DDAB mPEG-PCL
QDMP: Quercetin/ DDAB mPEG-PCL
EE: Encapsulation efficiency
DL: Drug loading
NTC: Non-template control
IC50: Half-maximal inhibitory concentration.
